# Potential for sylvatic and urban *Aedes* mosquitoes from Senegal to transmit the new emerging dengue serotypes 1, 3 and 4 in West Africa

**DOI:** 10.1371/journal.pntd.0007043

**Published:** 2019-02-13

**Authors:** Alioune Gaye, Eryu Wang, Nikos Vasilakis, Hilda Guzman, Diawo Diallo, Cheikh Talla, Yamar Ba, Ibrahima Dia, Scott C. Weaver, Mawlouth Diallo

**Affiliations:** 1 Unité d’Entomologie Médicale, Institut Pasteur de Dakar, Dakar, Senegal; 2 Institute for Human Infections and Immunity, Center for Tropical Diseases and Department of Microbiology and Immunology, University of Texas Medical Branch, Galveston, Texas; 3 Department of Pathology and Center for Biodefense and Emerging Infectious Diseases, University of Texas Medical Branch, Galveston, Texas, United States of America; 4 Epidemiological Infectious Disease Unit, Institut Pasteur de Dakar, Dakar, Senegal; University of Florida, UNITED STATES

## Abstract

Dengue fever (DEN) is the most common arboviral disease in the world and dengue virus (DENV) causes 390 million annual infections around the world, of which 240 million are inapparent and 96 million are symptomatic. During the past decade a changing epidemiological pattern has been observed in Africa, with DEN outbreaks reported in all regions. In Senegal, all DENV serotypes have been reported. These important changes in the epidemiological profile of DEN are occurring in a context where there is no qualified vaccine against DEN. Further there is significant gap of knowledge on the vector bionomics and transmission dynamics in the African region to effectively prevent and control epidemics. Except for DENV-2, few studies have been performed with serotypes 1, 3, and 4, so this study was undertaken to fill out this gap. We assessed the vector competence of *Aedes (Diceromyia) furcifer*, *Ae*. *(Diceromyia) taylori*, *Ae*. *(Stegomyia) luteocephalus*, sylvatic and urban *Ae*. *(Stegomyia) aegypti* populations from Senegal for DENV-1, DENV-3 and DENV-4 using experimental oral infection. Whole bodies and wings/legs were tested for DENV presence by cell culture assays and saliva samples were tested by real time RT-PCR to estimate infection, disseminated infection and transmission rates. Our results revealed a low capacity of sylvatic and urban *Aedes* mosquitoes from Senegal to transmit DENV-1, DENV-3 and DENV-4 and an impact of infection on their mortality. The highest potential transmission rate was 20% despite the high susceptibility and disseminated infection rates up to 93.7% for the 3 *Ae*. *aegypti* populations tested, and 84.6% for the sylvatic vectors *Ae*. *furcifer*, *Ae*. *taylori* and *Ae*. *luteocephalus*.

## Author summary

Dengue fever remains a major public health problem in all tropical regions of the world and causes 390 million infections each year. In Africa, while dengue outbreaks were rare during the last century, recurrent urban epidemic have been reported in all regions the last decade. Serotype 3, never reported in West Africa, caused major outbreaks in 2009 in several capital cities while serotype 2, usually confined to the forest cycle, spilled over into urban areas in Senegal and Mauritania in 2014–2015. These changes are occurring in a context where vector control remains the only effective approach to prevent and control epidemics. However, the design and the implementation of efficient vector control require an accurate knowledge of the vector bionomics and competence while such data are lacking in the African region. To fill out this gap we experimentally infected domestic and wild mosquitoes from Senegal to assess their vector competence for dengue serotypes 1, 3 and 4. Finally both domestic and wild Senegalese mosquitoes showed a low ability to transmit dengue viruses.

## Introduction

Dengue fever (DEN) is the most common arboviral disease in the world and is caused by four genetically distinct serotypes of virus (DENV-1, DENV-2, DENV-3, DENV-4) belonging to the genus *Flavivirus* of the family *Flaviviridae*. Among the 390 million annual infections estimated around the world, 240 million are inapparent and only 96 million are symptomatic [1]. Dengue fever causes a wide clinical spectrum similar for the four serotypes. The different clinical manifestations of DENV infection range from asymptomatic to several symptomatic forms ranging in severity from classical dengue fever, to Dengue Hemorrhagic Fever (DHF) and Dengue Shock Syndrome (DSS). Dengue viruses are transmitted to humans by mosquitoes of the genus *Aedes*, mainly by the peridomestic mosquito *Aedes aegypti aegypti* and secondarily by *Ae*. *albopictus* and other anthropophilic *Aedes* mosquitoes.

In Africa, the sylvatic circulation of DENV-2 appears to be predominant [2] in contrast to Asia and South America where endemic/epidemic DENV strains circulating in peridomestic cycles are most common, and a sylvatic, nonhuman primate-amplified enzootic cycle has not been identified except for in Malaysia. The first isolations of DENV-2 from naturally infected mosquitoes in Africa date to 1969 when two strains were isolated from Ibadan and Jos in Nigeria [3]. Thereafter, several epizootics of DENV-2 were reported through the periodic amplifications of the sylvatic cycle involving wild populations of mosquitoes and monkeys in several West African countries [4]. However, despite these frequent epizooties and the presence of the epidemic vector *Ae*. *aegypti* in all bioclimatic areas, only sporadic DEN cases were recorded in West Africa. This could be explained by the presence of *Aedes aegypti formosus*, the ancestral African sylvatic and zoophilic form that uses tree holes as its larval habitat. Indeed, both sub-species exist in Africa but the presence of *Aedes aegypti aegypti* (the domestic, highly anthropophilic and primarily endophilic subspecies) in West Africa remains debatable mainly because of the lack of reliable methods to distinguish the two subspecies. The first documented outbreak caused by DENV-2 in West Africa occurred in Burkina Faso in 1982 and was suspected to be triggered by an introduction from the east of an epidemic Seychelles strain [2].

Most African DEN outbreaks caused by DENV-2 have occurred in East Africa. The others DENV serotypes (1, 3 and 4) are only known from endemic-epidemic cycles in Africa with no evidence of enzootic circulation. Only DENV-1 has been found associated with *Ae*. *aegypti*. During the last century, DENV-1 epidemics were notified in South Africa in 1926–27, Sudan in 1984, and Nigeria in 1964 and 1975 while the unique DENV-3 outbreaks occurred in Mozambique in 1985 [5,6]. Serotype 4 was only reported in Senegal in contexts which still remains enigmatic [7]. Amarasinghe et al. 2011 [6] have presented an exhaustive review on dengue situation in Africa.

Over the last 2 decades a changing epidemiological pattern has been observed in Africa, with outbreaks of DEN reported in all regions and several cases exported to Europe [8].

DENV-2, responsible for several epidemics in East Africa (Somalia, Djibouti, Kenya and Tanzania) and usually circulating in a sylvatic cycle (between *Aedes* mosquitoes and non human primates) in West Africa, spilled over into urban areas in 2014–2015 in Senegal and Mauritania, Gabon in 2007, Angola in 2013 and Burkina Faso in 2016. Serotype 3 (DENV-3), never reported in Africa after its first emergence in 1985 in Mozambique, caused a major urban outbreak in 2009 in Cape Verde, Cote d’Ivoire, Gabon and Senegal. Since September 2017, Burkina Faso and Senegal face up to major urban outbreaks Ouagadougou and Louga respectively (S1 Table) [9,10,11,12,13,14,15,16,17,18,19,20,21,22,23]. In Senegal all DENV serotypes have been reported (S2 Table).

These important changes summarized above in the epidemiological African profile of DEN are occurring in a context where there is no vaccine against DENV recommended for all populations. Furthermore, there is a significant gap of knowledge on DENV vector bionomics and transmission dynamics in Africa to effectively prevent and control epidemics. The vector competence of mosquitoes associated with DENV in nature is poorly characterized. Except for DENV-2 [24,25] few studies have been performed with serotypes 1, 3, and 4 [26].

Following the 2009 Dakar DENV-3 epidemic, we initiated a vector competence study to evaluate the ability of *Ae*. *aegypti* populations from Dakar and Kedougou to transmit DENV-1 and -3, for which there is no evidence of enzootic, sylvatic circulation in Africa [27]; these two serotypes appear to circulate only in an endemic/epidemic cycle with peridomestic human amplification. Our prior results showed low susceptibility to DENV-3 but high infection and dissemination rates with DENV-1. However, the oral DENV doses used were low and transmission potential was not tested. Furthermore, only *Ae*. *aegypti* was tested and vector competence data for sylvatic vectors were generated for DENV-1, -3 and -4. Thereby the present study assessed the vector competence of Senegalese *Ae*. *aegypti*, *Ae*. *furcifer*, *Ae*. *taylori* and *Ae*. *luteocephalus* for DENV-1, -3 and -4.

## Materials and methods

### Ethics statement

The University of Texas Medical Branch (UTMB) Institutional Animal Care and Use Committee approved all experiments involving animal-derived cells/tissues/sera/samples under protocol 02-09-068. UTMB complies with all applicable regulatory provisions of the U.S. Department of Agriculture (USDA)-Animal Welfare Act; the National Institutes of Health (NIH), Office of Laboratory Animal Welfare-Public Health Service (PHS) Policy on Humane Care and Use of Laboratory Animals; the U.S Government Principles for the Utilization and Care of Vertebrate Animals Used in Research, Teaching, and Testing developed by the Interagency Research Animal Committee (IRAC), and other federal statutes and state regulations relating to animal research. The animal care and use program at UTMB conducts reviews involving animals in accordance with the *Guide for the Care and Use of Laboratory Animals* (2011) published by the National Research Council.

### Mosquito species

**Fig 1:**
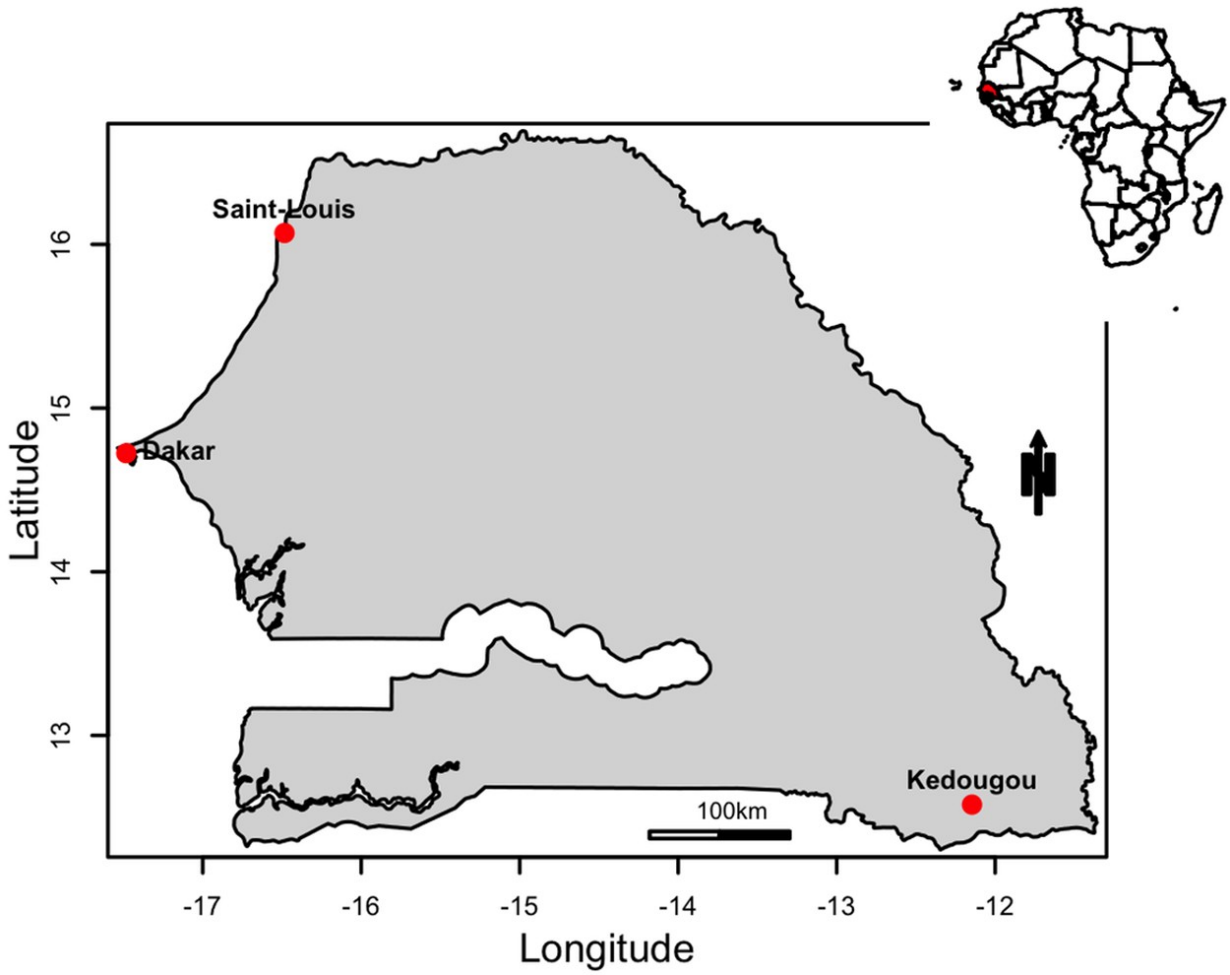
Map showing the three localities where mosquitoes were collected in Senegal for experimental infections with dengue viruses. This map was built using a shapefile from the free domain of the Geographic Information System (http://www.diva-gis.org) with the R software version 3.3.1 and the package maptools.

Mosquito species used in this study were collected from three Senegalese localities: Dakar, Saint Louis and Kedougou (Fig 1). The Table 1 describes the characteristics and geographic origins of *Ae*. *aegypti*, *Ae*. *furcifer*, *Ae*. *taylori*, and *Ae*. *luteocephalus* populations tested. The sylvatic *Ae*. *aegypti* population from Kedougou breeding in tree holes represented *Ae*. *aegypti formosus* morphologically characterised by the lack of pales scales on the first abdominal tergite and the urban populations from Dakar and Saint Louis breeding in artificial containers were consistent with *Ae*. *aegypti aegypti* contrariwise characterised by the presence of pales scales. These species were chosen based on their abundance, anthrophophilic behavior and association with DENV in nature. For each population, several larval habitats were sampled and immature stages were collected and reared in the laboratory. For *Ae*. *furcifer*, *Ae*. *taylori* and *Ae*. *luteocephalus* adult females were caught in a gallery forest at Kedougou and reared in the laboratory. Progeny of these populations were considered as the F1 generation that we used for experimental infections. Adult mosquitoes were maintained with a 10% sucrose solution at 27 °C, 75–80% relative humidity (RH), 12:12 h (Light:Dark) photoperiod.

**Table 1:** Mosquito species tested in this study.

Species	Source	Geographic position	Year of collection	Habitat	Gene-ration
***Ae*. *aegypti***	Dakar	17°28’24 W 14°43’29 N	2014	Domestic	F1
***Ae*. *aegypti***	Saint Louis	16°29’20 W 16°01’16 N	2014	Domestic	F1
***Ae*. *aegypti***	Kedougou	12°11’00 W 12°33’00 N	2014	Sylvatic	F1
***Ae*. *furcifer***	Kedougou	12°11’00 W 12°33’00 N	2014	Sylvatic	F1
***Ae*. *taylori***	Kedougou	12°11’00 W 12°33’00 N	2014	Sylvatic	F1
***Ae*. *luteocephalus***	Kedougou	12°11’00 W 12°33’00 N	2014	Sylvatic	F1

W: west, N: north

### Virus strains and preparation of stocks

Hosts origin, year of collection and passage histories of the virus strains used in this study are presented in Table 2. DENV-1, DENV-3 and DENV-4 strains obtained from the World Reference Center for Emerging Viruses and Arboviruses at the University of Texas Medical Branch in Galveston, Texas. For the *Ae*. *furcifer* experiment we used the DENV-4 strain from Haiti (Haiti 73), and DENV-3 strain from Barbados in the Caribbean region of North America (Carec 01–11828). For other mosquito species we used the following African strains: DENV-1 (SH 29177); DENV-3 strain (S-162 TvP-3622), and DENV-4 strain SH 38549.

**Table 2:** Dengue virus strains used for this study.

DENV strains	Reference	Host origin	Year of collection	Location	Passage history
DENV-1	SH 29177	Human	1979	Senegal (Bandia)	C6/36-3
DENV-3	Carec 01–11828	Human	2001	Barbados	C6/36-3
DENV-3	S-162 TvP-3622	Human	1993	Somalia	C6/36-2
DENV-4	SH 38549	Human	1983	Senegal (Dakar)	C6/36-3
DENV-4	Haiti 73	Human	1994	Haiti	C6/36-3

An additional passage on C6/36 cells was performed for each strain to obtain the viral stock used to infect mosquitoes. Cell lines were provided by the American Type Culture Collection (Manassas, Va.), and cultured in Gibco DMEM (Dulbecco’s Modified Eagle Medium), High glucose (Gibco Cat. No. 11965–092) supplemented with 10% fetal bovine serum (FBS; Atlanta Biologicals Cat. No. S11150) heat-inactivated in 56° C water bath for 60 min, penicillin-streptomycin (Gibco Cat. No. 15140–122) and 10% of Bacto Tryptose Phosphate Broth (Becton, USA). Virus in cell culture supernatants was concentrated using Millipore UFC910024 Amicon Ultra-15 Centrifugal Filter Concentrator with Ultracel 100 Regenerated Cellulose Membrane. Concentrated viruses were collected, aliquoted and frozen at -80°C, and used as viral stocks for mosquito infection. Virus stocks were titrated using the method focus forming assays and immunostaining described below.

### Focus forming assays and immunostaining

Virus titers for stocks and infectious blood meals after 1 hour of exposure to mosquitoes were determined by focus forming assays and immunostaining as described previously [28]. Briefly, Ten-fold serial dilutions of virus in MEM supplemented with 2% FBS and antibiotics (Invitrogen, Carlsbad, CA), were added in duplicate to confluent C6/36 cell monolayers attached to 24-well Costar (Corning, NY) plates, and incubated for 1 h with periodic gentle rocking to facilitate virus adsorption at 28 °C. Wells were then overlaid with 1 ml of 0.8% methylcellulose (Sigma-Aldrich, St. Louis) diluted in warm Optimem (Invitrogen) supplemented with 2% FBS, antibiotics and 1% (w/v) L-glutamine and incubated undisturbed for 4 days at 28 °C. Methylcellulose overlay was aspirated and cell monolayer rinsed once with phosphate buffered saline (PBS), pH 7.4 (Invitrogen) followed by fixation with a mixture of ice-cold acetone and methanol (1:1) solution and allowed to incubate for 30 min at room temperature (RT). Fixation solution was aspirated and plates were allowed to air dry. Plates were washed thrice with PBS supplemented with 3% FBS, followed by hour-long incubation with a dengue-specific hyperimmune mouse ascitic fluid. Mouse hyperimmune sera (MIAF) to DENV were prepared in adult mice; using 10% crude homogenates of DENV- infected newborn mouse brain in phosphate-buffered saline as the immunogen. The immunization schedule consisted of four intraperitoneal injections of antigen mixed with Freund’s adjuvant, given at weekly intervals. After the final immunization, mice were inoculated with sarcoma 180 cells, and the resulting immune ascitic fluids were collected. All animal work was done at UTMB under an IACUC approved animal use protocol (number 9505045). Plates were washed thrice followed by hour-long incubation with a secondary antibody, goat anti-mouse conjugated to horseradish peroxidase (HRP) (KPL, Gaithersburg, MD). Detection proceeded with the addition of aminoethylcarbazole (AEC) substrate (ENZO Life sciences, Farmingdale, CT) prepared according to vendor instructions.

### Oral infection of mosquitoes

Three- to 5-day-old F1 female mosquitoes were placed into 500 mL cardboard containers and sucrose-starved for 48 hours before being exposed to an infectious artificial blood meal (**Hemotek** Ltd, UK) using BALB/c mouse skins obtained from the University of Texas Medical Branch Animal Resource Center, as membranes. The blood meal contained a 33% volume of washed sheep erythrocytes and a 33% volume of a cell culture-derived virus stock supplemented with 21% FBS, and adenosine triphosphate (ATP) to a final concentration of 0.005 M as a phagostimulant, and sucrose at a final concentration of 10%. After feeding for up to 60 minutes, the remaining blood meal was kept at– 80 °C for virus titration using plaque assay then mosquitoes were cold-anaesthetized and fully engorged specimens were incubated with 10% sucrose at 27°±1°C, a relative humidity of 70–75% and 12:12 h (Light:Dark) photoperiod.

### Virus detection in mosquitoes

At 7 or 15 days post bloodmeal (dpbm), mosquitoes were cold-anaesthetized and their legs and wings were removed. The proboscis of each mosquito was then inserted into a capillary tube containing 1–2 μL of FBS for salivation for up to 30 min then expectorated saliva was collected into a tube containing 100 μL of DMEM supplemented with 5% FBS. Detection of DENV in the mosquito body but not the wings/legs indicated a non-disseminated infection (limited to the midgut), whereas the presence of virus in both the body and wings/legs indicated dissemination into the hemocoel. Mosquito bodies as well as wings/legs of infected bodies were tested for DENV after homogenization in 400 μl of MEM containing 5% of FBS, and centrifugation for 2 min at 11,500 x g at 4 °C to separate virus supernatant and debris. For each sample, 100 μl of supernatant were cultured in 24-well plates containing Vero cell monolayers and DENV was detected by focus forming assays and immunostaining described above, but without the ten-fold serial dilutions. So detection was limited to presence/absence revelation. Saliva of infected wings/legs were tested to detect DENV presence by real-time RT-PCR using an internal control of 10 no-infected mosquito saliva pooled together; 100 μl of each sample was used for RNA extraction using the QIAamp Viral RNA Extraction Kit (QIAgen, Heiden, Germany), according to the manufacturer’s protocol. Dengue virus RNAs extracted from mosquito saliva were amplified using Bio-Rad iTaq universal probes one-step kit (Cat#172–5141) following Manufacturer’s protocol. For detecting DENV-1 and DENV-3, forward primer (5’ATTAGAGAGCAGATCTCTG 3’), reverse primer (5’TGACACGCGGTTTC 3’), and Probe 5’/56-FAM/TCAATATGCTGAAACGCG/3BHQ_1/-3’ were used; for DENV-4, forward primer 5’AAT AGA GAG CAG ATC TCTG 3’ was used. The RT‐PCR was performed by Quant Studio 6 Flex instrument made from applied BioSystems by life technologies. The cycling conditions were RT step at 50.0 °C for 10 min, at 95.0 °C for 3 min, and 43 cycles of 15 s at 94.0 °C and 1 min at 55 °C.

### Impact of infection on mosquito longevity

During our experiment with *Ae*. *furcifer*, we observed 5 days after oral DENV exposure a high mortality rate. Based on this observation, we planned subsequent experiments to include a negative control cohort exposed to uninfected blood meals to assess the effect of DENV on mortality. The uninfected blood meals used as the negative control contained a 33% volume of washed sheep erythrocytes and 33% volume of cell culture media (Gibco DMEM, High glucose supplemented with 10% fetal bovine serum, penicillin-streptomycin and 10% of Bacto Tryptose Phosphate Broth) supplemented with 21% FBS, and adenosine triphosphate (ATP) to a final concentration of 0.005 M as a phagostimulant, and sucrose at a final concentration of 10%. The Table 3 showed the sample size for each virus strain and for each mosquito populations. These mosquitoes were monitored twice daily for mortality until 15 dpbm for *Ae*. *taylori*, *Ae*. *aegypti* from Kedougou and St. Louis and 20 dpbm for *Ae*. *aegypti* from Dakar, then surviving mosquitoes were tested for DENV infection as described above.

**Table 3:** Sample size for each virus strain and for each mosquito populations.

Mosquito species	Sample sizes		
	DENV-1	DENV-3	DENV-4
***Ae*. *aegypti aegypti* Dakar**	158	156	165
***Ae*. *aegypti formosus* Kedougou**	90	93	148
***Ae*. *aegypti aegypti* St. Louis**	129	165	128
***Ae*. *taylori***	41	57	62

### Data analysis

Infection (number of positive bodies/total number of engorged mosquitoes incubated and tested), disseminated infection (number of mosquitoes with positive wings-legs/ total number of engorged mosquitoes incubated and tested) and transmission (number of mosquitoes with infected saliva/ total number of engorged mosquitoes incubated and tested) rates were calculated for each species and each dpbm. The rates obtained were compared using Fisher’s exact test. For *Ae*. *aegypti* populations potential impact of the virus serotype, incubation and mosquito origin were estimated using beta regression model. A Wilcoxon test was performed to compare differences between survivals among groups. For all tests, differences were considered statistically significant at p < 0.05 using R v. 2.15.1 (R Foundation for Statistical Computing, Vienna, Austria) [29].

## Results

**Fig 2:**
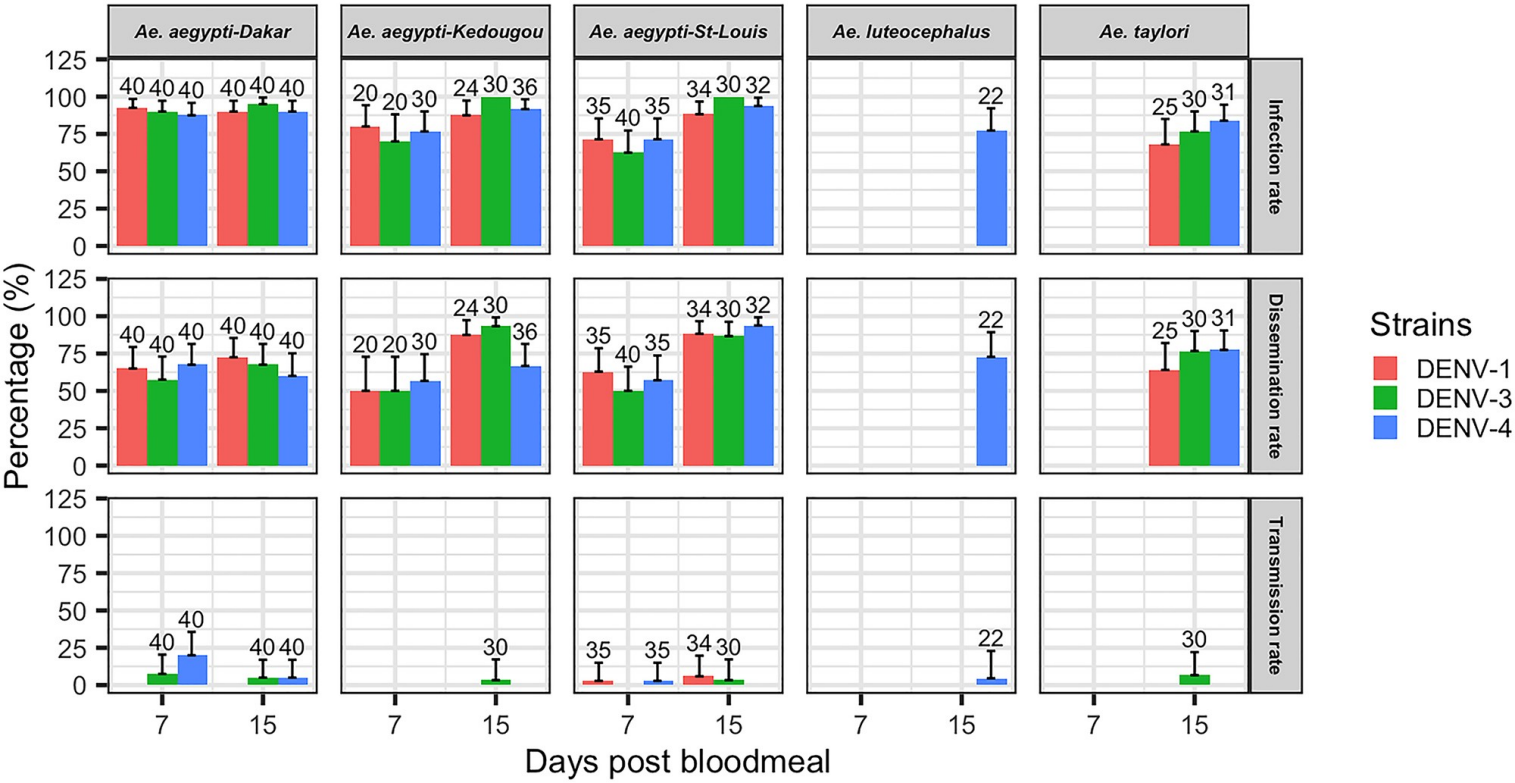
Infection, disseminated infection and transmission rates of four Senegalese *Aedes* mosquitoes orally exposed to different dengue serotypes at 7 and 15 days post bloodmeal. Error bars represent the upper limits of the 95% confidence intervals of infection, dissemination and transmission rates.

The titers of DENV stocks used ranged between 10^7^ and 10^8^ PFU/ml and the Table 4 presented the titers of the blood meals prepared from these stocks after 1-hour exposure at 37±1 °C for mosquitoes feeding in different days. These titers ranged between 1.2 x 10^6^ and 4.7 x 10^7^ PFU/ml. A total of 606 *Ae*. *aegytpi* (240 from Dakar, 206 from St. Louis and 160 from Kedougou), 86 *Ae*. *taylori*, 71 *Ae*. *furcifer* and 22 *Ae*. *luteocephalus* was tested after DENV exposure and incubation for 7 or 15 days. For *Ae*. *aegypti*, the minimum and maximum values of infection rates were 87.5–92.5% and 90–95% for the population from Dakar, 62.5–71.42% and 88.23–100% for the St. Louis population, and 70–80% and 87.5–100% for the Kedougou population, respectively, at 7 and 15 dpbm (Fig 2). Disseminated infection rates were 57.5–67.5% and 60–72.5% for the population from Dakar, 50–62.85% and 86.66–93.75% for the population from St. Louis and 50–56.66% and 66.66–93.33% for population from Kedougou respectively at 7 and 15 dpbm. While the infection and dissemination rates were high, the potential transmission (saliva infection) rates were globally low (0–20%), 0–5% for Dakar, 0–2.85% and 0–5.88% for St. Louis, and 0% and 0–3.33% for Kedougou, respectively at 7 and 15 dpbm.

**Table 4:** Titers of the infectious blood meal after 1 hour of exposure to the different mosquito species.

Populations of Mosquito species	Blood meal titers (PFU/mL)		
	DENV-1	DENV-3	DENV-4
***Ae*. *aegypti aegypti* Dakar**	4.7 x 10^7^	2.4 x 10^7^	1.2 x 10^6^
***Ae*. *aegypti formosus* Kedougou**	4.9 x 10^6^	3.5 x 10^6^	2.6 x 10^7^
***Ae*. *aegypti aegypti* St. Louis**	4.9 x 10^6^	3.5 x 10^6^	2.6 x 10^7^
***Ae*. *furcifer***	NA	3.1 x 10^6^	1.6 x 10^6^
***Ae*. *taylori***	4.9 x 10^6^	3.5 x 10^6^	2.6 x 10^7^
***Ae*. *luteocephalus***	NA	NA	1.4 x 10^6^

Results showed that all species were susceptible to disseminated infection with DENV-1, -3 and -4 (Fig 2 and S1 Fig). *Ae*. *aegytpi* population from Dakar showed higher infection rates (IR) than populations from St. Louis and Kedougou for all 3 dengue serotypes at 7 dpbm. However, differences were significant only between the Dakar and St. Louis populations for DENV-1 (Fisher’s exact test: p = 0.01) and DENV-3 (Fisher’s exact test: p = 0.003). Infection rates of *Ae*. *aegypti* populations increased significantly between 7 and 15 dpbm for all 3 serotypes except for the population from Dakar. At 15 dpbm, IRs of the 3 populations did not differ significantly (Fisher’s exact test: p > 0.05). For all *Ae*. *aegypti* populations, IRs with DENV-3 were higher than those obtained with DENV-1 and DENV-4. However, the difference was statistically significant only for *Ae*. *aegypti* from Kedougou when we compare IR obtained with DENV-3 versus DENV-1 (Fisher’s exact test: p = 0.04). The minimum and maximum values of disseminated infection rates of the 3 populations of *Ae*. *aegypti* were 50–65% for DENV-1, 50–57.5% for DENV-3 and 56.66–67.5% for DENV-4 and were statistically comparable at 7 dpbm (Fisher’s exact test: p > 0.05), while at 15 dpbm *Ae*. *aegypti* from St. Louis showed significantly higher DIR than populations from Dakar (Fisher’s exact test: p = 0.001) and Kedougou (Fisher’s exact test: p = 0.005) for DENV-4. With *Ae*. *aegypti* populations from Kedougou and St. Louis the IRs and DIRs increased between 7 and 15 dpbm, however the population from Dakar were susceptible to infection and developed disseminated infection with same rates at 7 and 15 dpbm.

Among the sylvatic vectors, *Ae*. *furcifer* showed the highest IRs for DENV-3 and DENV-4 but differences were not significant (Fisher’s exact test: p > 0.05). No significant difference was observed for DENV-4 infection and dissemination among the three species despite the higher IR with *Ae*. *furcifer* and lower with *Ae*. *luteocephalus*.

**Fig 3:**
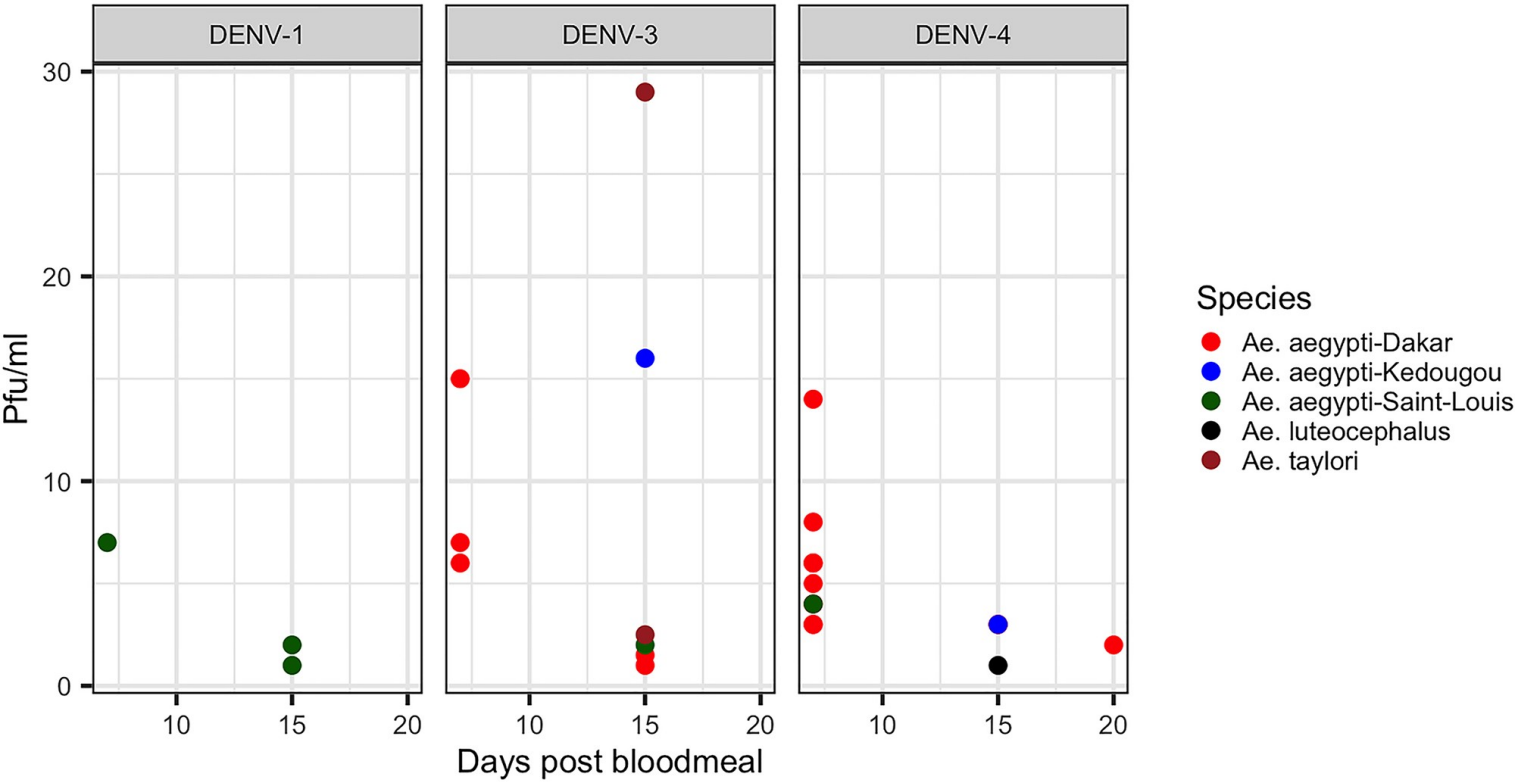
Virus titers of infected mosquito saliva at 7 and 15 dpbm (PFU/ml).

The Fig 3 shows titers of the infected saliva. Globally we observed mainly for *Ae*. *aegypti* a decreasing of titer between 7 dpbm and 15 dpbm. The highest titer (29 PFU/ml) was observed with *Ae*. *taylori*. For *Ae*. *aegypti* highest titers were 15 and 16 PFU/ml for Dakar and Kedougou mosquito strains at 7 and 15 dpbm respectively.

The regression model did not reveal an effect of the virus serotype on the infection rates of *Ae*. *aegypti* populations (Table 5). However, odds ratio of mosquito strain from Saint-Louis versus mosquito strains from Dakar, decreases significantly by a factor of 0.4 (p<0.001) while relative proportion of infected mosquitoes increases by a factor of 3.5 at 15 dpbm compared to 7 dpbm (p<0.001).

**Table 5:** Beta regression model estimating relationship between infection and virus serotype, incubation and mosquito origin.

	Infection			Dissemination			Transmission		
	OR	CI	P-value	OR	CI	P-value	OR	CI	P-value
**Geographic Origin**									
Dakar	1			1			1		
Kedougou	0.55	0.29–1	0.06	1.14	0.7–1.8	0.5	0.65	0.03–4.47	0.7
Saint-Louis	0.41	0.23–0.7	<0.001	1.44	0.96–2.2	0.08	0.27	0.04–1	0.09
**Days post Blood meal**									
Day7	1			1			1		
Day15	3.5	2–6	<0.001	2.59	1.8–3.7	<0.001	0.55	0.19–1.4	0.2
**Virus serotypes**									
DENV-1	1			1			1		
DENV-3	1.1	0.6–2	0.7	0.8	0.5–1.2	0.3	0.45	0.05–3.25	0.4
DENV-4	1	0.56–1.78	0.9	0.79	0.5–1.2	0.2	0.80	0.1–5.12	0.8

OR: Odd Ratio; CI: Confidence Interval

For the dissemination rates, no effects of the virus serotype and mosquito origin were observed. The incubation period was the unique parameter affecting the dissemination with an odds ratio at 15 dpbm increasing by a factor of 2.59 compared to 7 dpbm.

No statistically significant relationship between transmission rate and mosquito origin, virus strains and dpbm.

**Fig 4:**
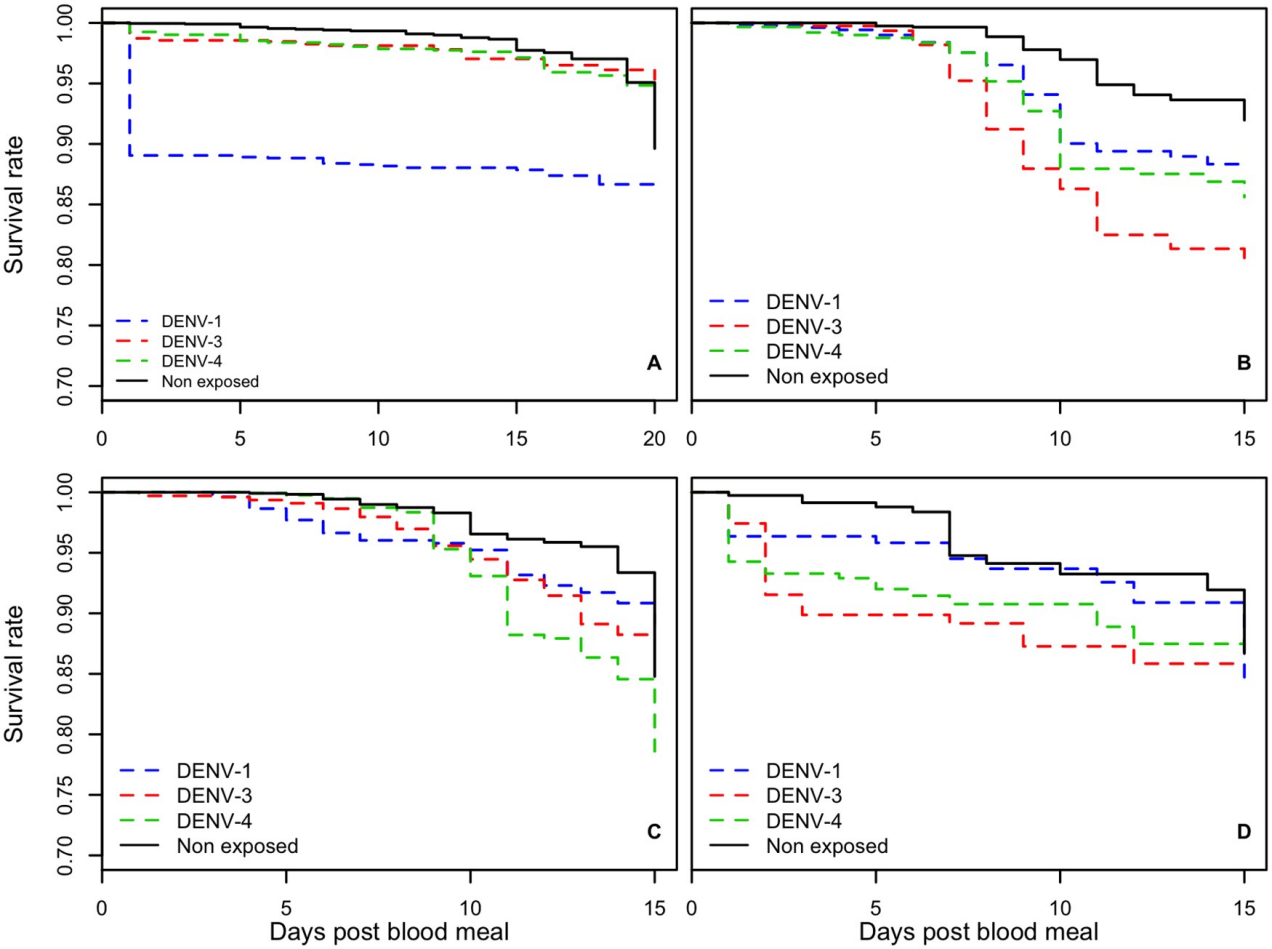
Daily changes in survival after exposure of *Ae*. *aegypti* populations from Dakar (A), from St. Louis (B) and from Kedougou (C) and *Aedes taylori* (D) to infected blood meals with dengue viruses 1, 3 and 4 and uninfected blood used as control (Non exposed).

When we compared survival of *Ae*. *aegypti* mosquitoes from Dakar exposed to DENV-1, -3 and -4 with infection rates of 92.68, 93.02 and 91.66%, respectively, to that of the unexposed control group, globally the difference was statistically significant (Wilcoxon test: p <0.001) (Fig 4A). For *Ae*. *aegypti* mosquitoes from St. Louis exposed to DENV-1, -3 and -4 with infection rates of 88.23, 100 and 93.75% respectively compared to unexposed group (Fig 4B), significantly higher mortality was observed (Wilcoxon test: p = 2.57x10^-12^). For *Ae*. *aegypti* from Kedougou (Fig 4C) exposed to DENV-1, -3, -4 with infection rates of 87.5, 100, 91.66%, respectively, mortality was also significantly higher than that of the negative controls (Wilcoxon test: p = 0.0009). The difference was also overall significant for *Ae*. *taylori* (Wilcoxon test: p = 0.01) (Fig 4D), which showed infection rates of 68, 76.66, and 83.87% for DENV-1, DENV-3 and DENV-4 respectively.

For *Ae*. *aegypti* from Kedougou and *Ae*. *taylori*, our analysis did not reveal any effect between DENV serotypes and the mosquito population survival rate (p = 0.344 and p = 0.378 respectively). But survivals were significantly different between DENV serotypes for *Ae*. *aegypti* Dakar (p <0.001) and *Ae*. *aegypti* Saint Louis (p = 0.002). For *Ae*. *aegypti* from Dakar the DENV-1 induced the highest mortality (Wilcoxon test: p<0.001).

Kaplan–Meier survival curves are shown that mortality was higher for *Ae*. *aegypti* from Dakar exposed to DENV versus unexposed. Also, survival of this population was more affected by DENV-1 than by DENV-4 and DENV-3.

Our results showed that DENV infection also affected the survival of *Ae*. *aegypti* from St. Louis. Survival of mosquitoes exposed to all three DENV was reduced compared to negative controls from the 6^th^ dpbm.

*Ae*. *aegypti* from Kedougou and exposed to DENV-4 survived better until 9 dpbm, then mortality increased highly compared to controls from the 11th dpbm. Mosquitoes exposed to DENV-1 had reduced survival early compared to DENV-3 and DENV-4. From the 11^th^ dpbm, survival of *Ae*. *aegypti* from Kedougou was significantly affected by all three DENV serotypes.

Survival of *Ae*. *taylori* exposed to DENV-4 or -3 was significantly lower than controls at all dpbm, but there was no significant difference between DENV serotypes. While mosquitoes exposed to DENV-1 showed reduced survival for the first 7 dpbm, no significant difference was observed later.

## Discussion

Our study provides important information on the vector competence of both sylvatic and domestic populations of *Ae*. *aegypti* and three sylvatic species of *Aedes* while some aspect could be considered as minor limitations. First, due to limited number of specimens available we were not able to test *Ae*. *furcifer* with the African DENV strains after an early experiment performed with DENV-4 and DENV-3 strains respectively from Haiti and Barbados. For the same reason we also limited the experiment with *Ae*. *furcifer*, *Ae*. *taylori* and *Ae*. *luteocephalus* at 15 dpbm only. Furthermore, only the RT-PCR was used to detect DENV genomes whether infectious particles or not for 2 reasons: i) the purpose of this article is to show the competence of the vector and we have been focused on the detection of DENV in the different compartments of the mosquitoes and in the saliva. As we have shown that the virus reached the saliva, it implies that the vector is competent ii) In our experience with other viruses, (West Nile, Usutu), we have noticed that RT-PCR and infectious viral particles are generally very consistent and concordant in their conclusions and trends [30,31]. Such a trend has also been confirmed on C6/36 cells for other virus [32]. Despite the relevance of the capillary feeding method for virus transmission assessment there is no proof of salivation activity for each tested individual. However, as the use of animals has many limitations, it is currently the best alternative technique [33,34], successfully used over years with different media like defibrinated blood [35], mineral or immersion oils [36,37], foetal bovine serum [38] to measure virus transmission. One way to assess the presence of saliva could be the detection of saliva components like protein or carbohydrates. Such an approach will require, however, a larger volume of media for saliva collection to achieve the different analysis without compromising virus detection. During our experiments, no DENV-susceptible laboratory mosquito strain was used as control. In our knowledge, there is no unique mosquito strain that can serve as a single control for each of the DENV serotypes and genotypes. Beyond the current scope of the study, future experiments would take into account this aspect by integrating at least one laboratory susceptible mosquito strain for each DENV serotype.

Our results showed high infection and disseminated infection rates with DENV serotypes 1, 3 and 4, both for *Ae*. *aegypti* populations and for the sylvatic mosquito vectors *Ae*. *furcifer*, *Ae*. *taylori* and *Ae*. *luteocephalus*. IRs with *Ae*. *aegypti* population from Dakar reached their maximum values as early as 7 dpi, while for *Ae*. *aegypti* from Kedougou and St. Louis, IRs increased between 7 dpbm and 15 dpbm to reach their maximum values later. *Ae*. *aegypti* mosquitoes from Dakar develop DENV infection earlier than populations from Kedougou and St. Louis and this could be explained by highest blood meal titer for *Ae*. *aegypti aegypti* Dakar than others populations. However differences were not significant between populations from Dakar and Kedougou for DENV-1 and -3, as observed earlier [27], but IRs and DIRs were higher in the present study. This could be explained by difference of virus strains and especially by high oral virus doses (10^6^–10^7^ PFU/ml) compared to those used previously (10^3^–10^4^ MID_50_/ml (Mice Infectious Dose 50)) with different *Ae*. *aegypti* populations from Thailand [39]. Moreover *Ae*. *aegypti* populations from western (Burkina Faso: Koro, Bobo and Kari mosquito strains) and eastern (Kenya: Rabai and Shimba Hills mosquito strains) Africa showed lower rates despite the same viral titers (107.3–10^8.1^ MID_50_/ml). Similarly in many DEN-endemic countries infection and dissemination rates obtained [40] were lower than those showed by this study.

Comparisons between serotypes show that Senegalese *Ae*. *aegypti* were more susceptibility to DENV-3 than to other serotypes. Also, DENV-3 was detected in the saliva of all three populations; this could explain recent DENV-3 outbreaks in many African countries (S1 Table). The St. Louis population showed transmission potential for all three DENVs even if TRs were low, suggesting a salivary gland barrier within some *Ae*. *aegypti* populations. However, the population from Dakar showed higher TRs than other populations and at 7 dpbm these rates for DENV-4 reached 20% of total engorged mosquitoes and 7.5% for DENV-3. These transmission rates observed just a week after mosquito infection may explain the Dakar DENV-3 epidemic like the Cape Verde *Ae*. *aegypti* population transmitting during the 2009 outbreak while it showed infection rates of 0% at 7 dpbm and until 10 dpbm the transmission rates did not exceed 20% of only mosquitoes which disseminated the infection [41]. The potential transmission rates obtained with DENV-4 show that even if a large outbreak has not been reported, the risk of a DENV-4 epidemic is present in Dakar and St. Louis because, as noted before, even with low transmission rates a vector can cause epidemics based on its abundance, density, survival and human feeding frequency [42].

Regarding enzootic, sylvatic vectors, infection and dissemination rates were relatively high for the different serotypes tested (Fig 2 and S1 Fig). The same population of *Ae*. *furcifer*, with different DENV-2 strains, showed similar IRs (26 to 97%) and DIRs (17 to 75%) to the rates we obtained [24]. But in our study we did not detect transmission potential by *Ae*. *furcifer*. The decreasing of viral titers in mosquito saliva observed at least in *Aedes aegypti* between 7 and 15 dpbm may explain the low transmission rates obtained.

Vectorial capacity is the efficiency of a vector in the transmission of a pathogen due to the combined effects of many factors, both intrinsic and extrinsic. The mortality rate is an important component [43,44,45]. Even if vectors become infected after taking an infectious blood meal, if they fail to survive to bite another host, the potential for transmission by this population is low. As a result, changes in the mosquito mortality rate would directly affect transmission of the pathogen. In our study we found that the exposure of *Ae*. *aegypti* from Dakar, Kedougou and St. Louis to the 3 DENV serotypes significantly increases mortality compared to negative control cohorts exposed to uninfected blood meals. Adverse effects on the fitness of *Ae*. *aegypti* due to DENV infection were also reported previously [46]. We also found increased mortality of infected *Ae*. *taylori* mosquitoes. Several other studies showed effects of other arboviruses on the survival of mosquito vectors [47,48].

Our results showed that for all 3 populations of *Ae*. *aegypti*, DENV-1 exposure affects mosquito survival. However, for the *Ae*. *taylori* population, after 7 dpbm we no longer detected an effect on survival of mosquitoes with DENV-1 infection. This absence of effect of DENV-1 infection on *Ae*. *taylori* is surprising because this species is not normally adapted to this DENV serotype, which is not known to circulate in the forest galleries frequented by *Ae*. *taylori* in Africa [49]. Indeed, only DENV-2 has been shown to circulate regularly in a sylvatic cycle in southeastern Senegal in the Kedougou region [7,50]. In summary, our results indicate that DENV-4 exposure did not affect survival of *Ae*. *aegypti* from Kedougou before 9 dpbm but it affected early the survival of the *Ae*. *aegypti* populations from Dakar and Saint Louis and *Ae*. *taylori*. DENV-3 caused high mortality in all mosquito populations tested, mainly in *Ae*. *taylori* and *Ae*. *aegypti* from Dakar. Survival was been most affected by DENV-1 which showed the highest potential transmission rates.

## Supporting information

S1 Table Dengue outbreaks in Africa in the last two decades. (DOC)

S2 Table Dengue in Senegal. (DOC)

S1 Fig Infection, disseminated infection and transmission rates of *Aedes furcifer* orally exposed to DENV-3 and DENV-4 viruses from Barbados and Haiti at 15 days post bloodmeal. Error bars represent the upper limits of the 95% confidence intervals of infection and dissemination rates.
(TIF)
