# Supplementary material for: Potential for sylvatic and urban Aedes mosquitoes from Senegal to transmit the new emerging dengue serotypes 1, 3 and 4 in West Africa
Source: PLoS Negl Trop Dis. 2019 Feb 13;13(2):e0007043. doi: 10.1371/journal.pntd.0007043 (PMC6373929; doi:10.1371/journal.pntd.0007043)
Supplement: S1 Table — (DOC) [file pntd.0007043.s001.doc]

S1 Table. Dengue outbreaks in Africa in the last two decades.

| **Year** | **Country** | **Location** | **Cases** | **Deaths** | **Serotype** | **References** |
| --- | --- | --- | --- | --- | --- | --- |
| **2014-15** | Mozambique | Pemba and Nampula | 193 | 0 | 2 |  |
| **2014** | Tanzania | Kilosa, Dar es Salaam | 130 | 0 | 2 |  |
| **2013-14** | Kenya | Mombassa | 210 | 0 | 1,2, 3 |  |
| **2013** | Ethiopia | Dire Dawa | 11,409 | 0 | 2 |  |
| **2004-05** | Sudan | South Kordofan | 312 | 0 | 3 |  |
| **2010** | Sudan | Port Sudan | 3,765 | 10 |  |  |
| **2012-13** | Sudan | Red Sea | 738 | 6 |  | WHO sitrep June 2014 |
| **2012-13** | Somalia | Mogadishu | 107 | 3 | 1,2,3 |  |
| **2013-14** | Angola | Luanda | 313 | 1 | 1 |  |
| **2007** | Gabon | Cocobeach, Libreville, Ntoum, Kango, Mitzic, Oyem, Minvoul | 54 | 0 | 2 |  |
| **2010** | Gabon | Cocobeach, Libreville, Kango, Ntoum, Mitzic, Minvoul, Lambaréné, Ndjolé, Lastourville, Koulamoutou, Moandra, Okondja Franceville | 407 | 0 | 1, 2, 3 |  |
| **2013** | Burkina Faso | Ouagadougou | 24 | 0 | 3 |  |
| **2016** | Burkina Faso | Ouagadougou | 1,327 | 20 | 2, 3 |  |
| **2017** | Burkina Faso | Ouagadougou | 8,450 | 29 | 1,2,3 |  |
| **2009** | Senegal | Dakar | 196 | 1 | 3 |  |
| **2014-15** | Senegal | Dakar, Mbour, Touba | 42 | 0 | 2 | Unpublished |
| **2017** | Senegal | Louga, Dakar, Fatick, Thies | 138 | 0 | 1 | Unpublished |
| **2014-15** | Mauritania | Nouakchott | 43 | 0 | 2 | Unpublished |
| **2017** | Mauritania | Nouakchott | 36 | 0 | 1 | Unpublished |
| **2008** | Cote d’Ivoire | Abidjan | 21 | 0 | 3 |  |
| **2008** | Mali | Sadiola, Bamako | 70 | 2 | 2 |  |
| **2009** | Cape Verde | All islands | 21 313 | 6 | 3 |  |
