# Supplementary material for: Potential for sylvatic and urban Aedes mosquitoes from Senegal to transmit the new emerging dengue serotypes 1, 3 and 4 in West Africa
Source: PLoS Negl Trop Dis. 2019 Feb 13;13(2):e0007043. doi: 10.1371/journal.pntd.0007043 (PMC6373929; doi:10.1371/journal.pntd.0007043)
Supplement: S2 Table — (DOC) [file pntd.0007043.s002.doc]

S2 Table. Dengue in Senegal.

| **Year** | **Location** | **Serotype** | **Diagnostic** |
| --- | --- | --- | --- |
| **1970** | Bandia | 2 | Isolation (1) |
| **1979** | Bandia | 1 | Isolation (2) / IgM (2) |
| **1979-80** | Kaolack | 1 | IgM (42.8% of children) |
| **1980-82** | Bandia | 1 | IgM (21.5% of children) |
| **1981-82** | Mekhe | 1 | IgM |
| **1981-82** | Touba | 1 | IgM |
| **1980-1984** | Kedougou | 2 | Isolation |
| **1981** | Kedougou | 2 | IgM (11% of children) |
| **1982-1985** | Kedougou | 2 | IgM |
| **1983** | Dakar/Casamance | 4 | Isolation |
| **1988** | Kedougou | 2 | IgM (4.6-5.7%) |
| **1990** | Kedougou | 2 | Isolation (2) |
| **1991** | Kedougou | 2 | IgM (0.8%) |
| **2008** | Kedougou |  | IgM (19) |
| **2009** | Dakar | 3 | Isolation (196) |
| **2014-2015** | Dakar/Mbour/Touba | 2 | isolation |
