# Supplementary figures and images for: Potential for sylvatic and urban Aedes mosquitoes from Senegal to transmit the new emerging dengue serotypes 1, 3 and 4 in West Africa
Source: PLoS Negl Trop Dis. 2019 Feb 13;13(2):e0007043. doi: 10.1371/journal.pntd.0007043 (PMC6373929; doi:10.1371/journal.pntd.0007043)

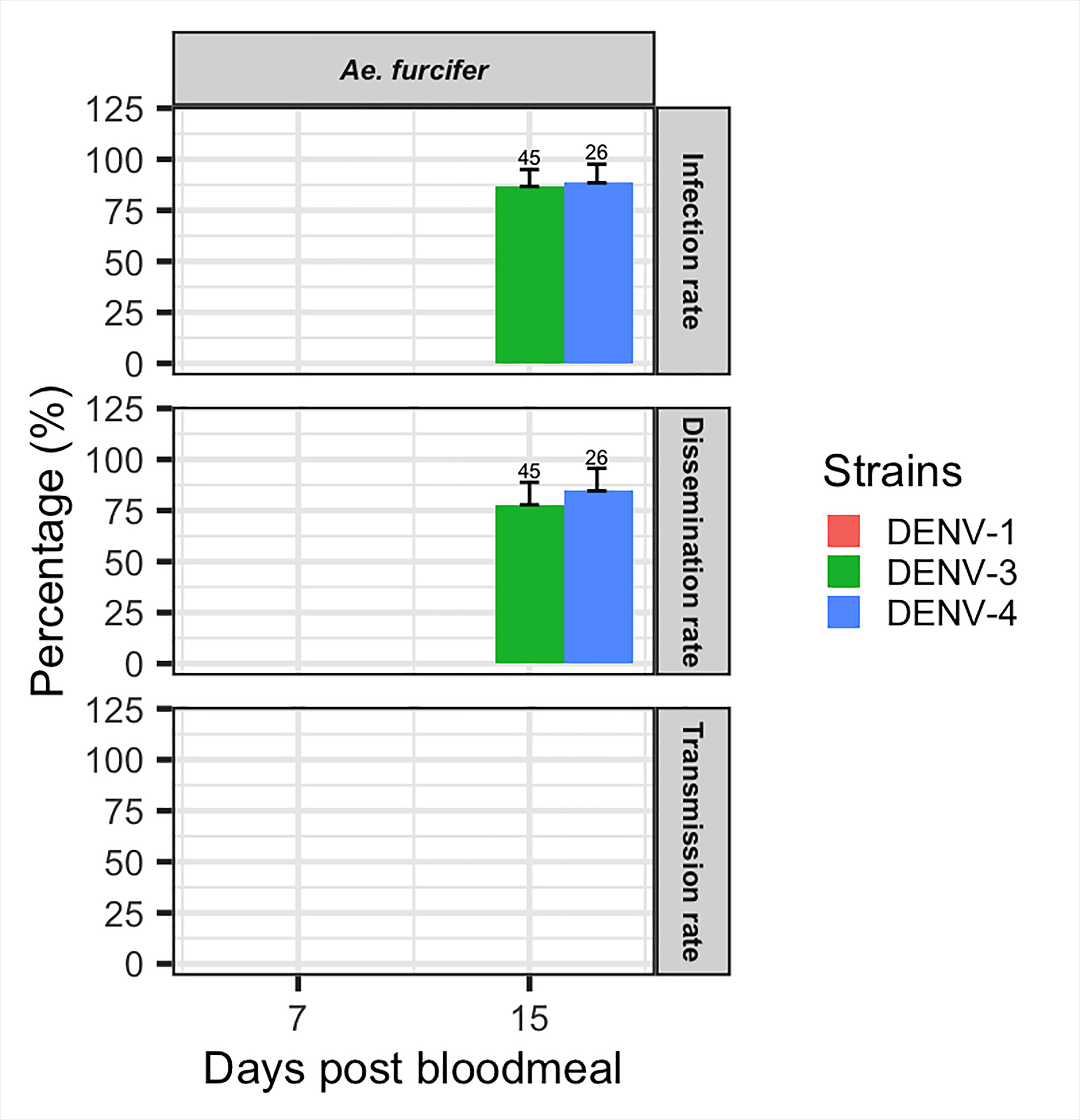

Supplement: S1 Fig — Error bars represent the upper limits of the 95% confidence intervals of infection and dissemination rates. (TIF) [file pntd.0007043.s003.tif]
